# Impaired Neonatal Outcome after Emergency Cerclage Adds Controversy to Prolongation of Pregnancy

**DOI:** 10.1371/journal.pone.0129104

**Published:** 2015-06-29

**Authors:** Ruben-J. Kuon, Hannes Hudalla, Christoph Seitz, Stephanie Hertler, Stephanie Gawlik, Herbert Fluhr, Hans-Jürgen Gausepohl, Christof Sohn, Johannes Pöschl, Holger Maul

**Affiliations:** 1 Department of Obstetrics and Gynecology, Heidelberg University Hospital, 69120, Heidelberg, Germany; 2 Department of Neonatology, Heidelberg University Hospital, 69120, Heidelberg, Germany; 3 Department of Obstetrics and Gynecology, Kath. Marienkrankenhaus, 22087, Hamburg, Germany; The Ohio State Unversity, UNITED STATES

## Abstract

**Objective:**

Emergency cervical cerclage is one of the treatment options for the reduction of preterm birth. The aim of this study is to assess neonatal outcome after cerclage with special focus on adverse effects in very low birth weight infants.

**Study Design:**

Retrospective cohort study. Classification of cerclages in history-indicated (HIC, n = 38), ultrasound-indicated (UIC, n = 29) and emergency/ physical examination-indicated (PEIC, n = 33) cerclage. Descriptive analysis of pregnancy and neonatal outcome (admission to NICU, duration of hospitalization, respiratory outcome (intubation, CPAP, FiO_2_max), neonatal complications (ROP, IVH)). Statistical comparison of perinatal parameters and outcome of neonates <1500 g after cerclage with a birth weight matched control group.

**Results:**

Neonates <1500 g after PEIC show significantly impaired outcome, i.e. prolonged respiratory support (total ventilation in days, CPAP, FiO_2_max) and higher rates of neonatal complications (IVH ≥ II, ROP ≥ 2). Placental pathologic evaluation revealed a significantly higher rate of chorioamnionitis (CAM) after PEIC. Neonates <1500 g after UIC or HIC show no significant difference in neonatal complications or CAM.

**Conclusions:**

In our study PEIC is associated with adverse neonatal outcome in infants <1500 g. The high incidence of CAM indicates a potential inflammatory factor in the pathogenesis. Large well-designed RCTs are required to give conclusive answers to the question whether to prolong or to deliver.

## Introduction

Preterm birth is a severe pregnancy complication as the impact on the future life of the newborn is dramatic and associated with many severe health problems [1]. Additionally, preterm birth is the single most important cause of perinatal death in North America, Europe, and particularly in low resource countries [1]. Despite all efforts, intensive research and progress in obstetrical care the problem of preterm births is increasing and affects about 5% to 11% of live births [1,2].

At the end of pregnancy the cervix progressively softens and shortens, which is mainly caused by a degradation of collagen fibrils and can be monitored by transvaginal ultrasound. Late miscarriage and preterm birth occur when these changes happen too early. A possible treatment option for preterm cervical changes is cervical cerclage [3]. The term cerclage subsumes all surgical procedures to reinforce the cervix with the help of sutures, wires or synthetic tapes. There are three different indications for cerclage: The history-indicated cerclage (HIC = prophylactic/elective) in women with previous second trimester loss or preterm birth, the ultrasound-indicated cerclage (UIC = therapeutic/salvage) in women with a short cervical length in the current pregnancy generally assessed by transvaginal ultrasound, and the physical examination-indicated cerclage (PEIC = emergency/rescue) in women with threatening miscarriage (bulging membranes assessed either digitally or by speculum examination) [4]. Possible side effects include uterine contractions and bleeding which might result in miscarriage or preterm birth [4].

It has to be taken into consideration that infections are well-established factors of preterm cervical shortening, contractions and dilation and therefore “trapping” the infection represents another potential adverse effect. The diagnosis of intrauterine infection or inflammation is difficult as we lack reliable clinical or biochemical indicators [5]. Maternal C-reactive protein (CRP) is no accurate predictor of clinical or histological chorioamnionitis (CAM) [5,6]. An intrauterine inflammation, which can also occur without rupture of membranes and without the presence of microbes in the amniotic fluid, has been shown to be associated with maternal morbidity and impaired neonatal outcome such as increased risk of cerebral palsy and cystic periventricular leukomalacia [5,7,8].

Performing a cerclage has been shown to serve as a valid procedure to reduce the incidence of preterm birth without significant reduction in neonatal morbidity [4]. A matter of controversy remains, whether the prolongation of pregnancy makes up for adverse effects of cerclage and eventually leads to improved neonatal outcome.

The goal of this study is to assess neonatal outcome after cerclage with special focus on adverse effects in very low birth weight infants (VLBW, <1500 g). We focus on preterm babies <1500 grams as they are at increased risk of neonatal morbidity and mortality with high medical relevance [9]. Further, we hypothesize that “trapping” an inflammatory process via a cerclage may cause adverse neonatal outcome. The VLBW subgroup represents a population with high susceptibility to inflammation and is thus most likely to show significant differences in perinatal outcome [9]. In contrast to many other studies we expand the evaluation period of neonatal outcome to the total time of primary neonatal hospitalization until discharge [4,10].

## Materials and Methods

This retrospective cohort study includes all patients who were treated by cerclage in the Department of Obstetrics and Gynecology at the University Clinic Heidelberg between 1999 and 2006. The study was approved by the Heidelberg Medical Faculty Ethics Committee. The ethics committee vote 370/2006 allows for anonymous retrospective analysis of patient data for this project. All patient information was anonymized and de-identified prior to analysis.

The standard technique at Heidelberg University was Mc Donald cerclage. Patient data of mothers and neonates was collected retrospectively using the medical records and our electronic patient management system (ISH-med 4.72; SAP, Walldorf, Germany; Neodat 5.1450.1; Paedsoft, Tübingen, Germany). Cases missing relevant information such as gestational age (GA) of treatment, unclear or undefined indication or missing outcome data were not included.

Inclusion criteria: The different types of cerclage were defined as: History-indicated cerclage (HIC), in pregnancies with a history of ≥ 1 preterm birth < 34 gestational weeks and/ or second trimester loss; Ultrasound-indicated cerclage (UIC), transvaginal ultrasound cervical length < 25 mm before gestational week 24 during current pregnancy; Physical examination-indicated cerclage (PEIC), cervical dilatation ≥ 2 cm and/ or bulging membranes. Both singleton and multiple pregnancies were included.

Exclusion criteria at time of cerclage placement: Patients with preterm rupture of membranes, vaginal bleeding, regular uterine contractions (> 3 contractions/ 30 minutes, tocography), clinical (temperature > 100.4°F, uterine tenderness and pain) or biochemical (C-reactive protein > 5 mg/l, leukocytes > 10.000/ μl) signs of infection, including testing for active vaginal infection by swab test, as well as chronic maternal infections (HIV, Hepatitis B and C) were excluded from this study.

For statistical analysis of neonatal outcome of infants <1500 g a control group was generated with neonates born in the study period (1999–2006) without a history of maternal cerclage (n = 652). We performed frequency matching on birth weight categories (extremely low birth weight infants, ELBW (<1000 g) and VLBW (1000–1500 g)), in order to adjust for birth weight in all three subgroups and the control group (resulting in: n_control_ = 373; n_PEIC_ = 20; n_UIC_ = 10; n_HIC_ = 12). Triplet pregnancies were excluded for this analysis. The fraction of twins was equal in all three subgroups and the control group (resulting in: multiples n_control_ = 40, 10.7%; n_PEIC_ = 2, 10.0%; n_UIC_ = 1, 8.3%; n_HIC_ = 1, 10%).

Placental histopathological examination was conducted by the Department of Pathology at Heidelberg University Hospital. CAM was diagnosed according to histopathologic evidence of inflammation, i.e., the presence of neutrophilic infiltrate in the amniotic and chorionic membranes.

Description of the variables statistically assessed in the subgroup of neonates <1500 g: Maternal parameters: rate of preterm premature rupture of membranes (PPROM), reasons for preterm delivery, delivery mode. Infection-associated parameters: CRP levels and white blood cell count (WBC) at delivery, placental pathologic evaluation. Prenatal treatment: antibiotic treatment, RDS prophylaxis (corticosteroids), tocolysis (fenoterol, i.v.). Neonatal parameters: birth weight, gestational age, APGAR scores, cord pH and base excess, clinical risk index for babies (CRIB score), respiratory parameters (duration of total ventilation / continuous positive airway pressure ventilation (CPAP), maximal fraction of inspired oxygen (FiO_2_max) and neonatal complications (intraventricular hemorrhage (IVH); retinopathy of prematurity (ROP)).

Statistical analysis was performed using R (R Core Team (2013). R: A language and environment for statistical computing. R Foundation for Statistical Computing, Vienna, Austria, http://www.R-project.org). Various statistical methods were used, including analysis of variance (ANOVA), Tukey’s test, Chi-squared test, Kruskal-Wallis test, Welch Two Sample t-test, Wilcoxon rank sum test with continuity correction and are referred to in the corresponding results section. Time-to-event analyses were calculated based on the corresponding Kaplan-Meier estimator and compared by log-rank-test. Bonferroni correction was applied to adjust p-values for multiple comparison.

## Results

In this study we identified 106 cases of cerclage (excluded: 5 patients received a cerclage in two different pregnancies–these cases were not included for statistical analysis as birth weight was > 1500 g; 6 patients delivered in a different clinic and no neonatal follow-up data was available) that were treated in our department and matched the criteria of inclusion (see above) resulting in 123 live born neonates (see descriptive maternal and neonatal outcome, Tables 1 and 2).

**Table 1:** Descriptive maternal characteristics and pregnancy outcome.

	Type of cerclage			
	HIC (n = 38)	UIC (n = 29)	PEIC (n = 33)	*P*
Age (y)	33 [25–42]	32 [26–38]	33 [24–40]	0.941
Gravidity	4 [1–7]	3 [0–4]	2 [0–5]	0.023
**Previous pregnancies**				
Second trimester loss	18 (47)	4 (14)	9 (27)	0.011
> 2 second trimester losses	11 (29)	2 (7)	2 (6)	<0.001
Previous preterm delivery	11 (29)	8 (28)	4 (12)	0.191
> 2 preterm deliveries	3 (9)	0	1 (3)	0.248
Term deliveries	9 (24)	8 (28)	10 (33)	0.819
**Previous cervical surgery**				
s/p conization	3 (7)	2 (8)	2 (6)	0.955
s/p cerclage	8 (22)	2 (8)	3 (9)	0.167
**Present pregnancy**				
Singleton	32 (84)	20 (69)	25 (76)	0.333
Twins	2 (5)	5 (17)	8 (24)	0.076
Triplets	4 (11)	4 (14)	0	0.104
GA at cerclage (w)	15 [12–16]	19 [17–24]	22 [19–24]	0.002
GA at delivery (w)	35 [20–41]	32 [24–41]	28 [20–41]	0.011
Cerclage to delivery interval (d)	131 [57–196]	95 [26–161]	43 [1–131]	<0.001

Table 1 shows maternal characteristics and pregnancy outcome for all included cases of cerclage. Data are shown as n (%) or median [range], y = years, s/p = status post, GA = gestational age, w = weeks, d = days.

**Table 2:** Descriptive neonatal outcome.

Outcome	Neonates after cerclage			
	HIC (n = 48)	UIC (n = 42)	PEIC (n = 33)	*P*
Live birth rate (%)	46 (95)	42 (100)	26 (80)	<0.001
**Birth weight** (g)	2093 [±1007] (1990) [1132–2965]	1917 [±899] (1858) [1221–2375]	1329 [±769] (1115) [700–1620]	<0.001
ELBW (< 1000 g)	11 (23)	8 (19)	14 (42)	0.043
- Of these < 750 g	5 (11)	6 (14)	12(36)	0.009
VLBW (1000 to < 1500 g)	3 (6)	8 (19)	6 (18)	0.150
LBW (1500 to < 2500 g)	15 (32)	17 (40)	11 (33)	0.640
≥ 2500 g	18 (38)	9 (21)	2 (6)	0.004
**Gestational age** (w)	33 [±5] (35) [28–37]	32 [±5] (32) [29–35]	29 [±5] (28) [24–31]	<0.001
Preterm (< 37)	30^a^ (64)	33 (79)	31 (94)	0.004
- Extremely preterm (< 28)	12 (26)	8 (19)	14 (42)	0.070
- Very preterm (28 to < 32)	6 (13)	6 (14)	11 (33)	0.041
- Moderate to late preterm (32 to < 37)	12 (26)	19 (45)	6 (18)	0.025
Term (≥ 37)	17 (36)	9 (21)	2 (6)	0.008
**Hospitalization of preterms (< 37 w)**				
Admission to NICU	26^a^ (87)	32 (97)	31 (100)	0.052
Total stay in neonatology (d)	73 [±49] (71) [28–120]	51 [±36] (34) [26–78]	86 [±56] (87) [35–121]	0.013
**Respiratory outcome**				
Respiratory support^b^	15 (60)	18 (56)	22 (71)	0.007
- Duration of therapy (d)	13 [±21] (2) [0–12]	12 [±21] (1) [0–10]	23 [±26] (15) [0–40]	0.012
Mechanical ventilation	11 (44)	6 (19)	17 (55)	0.001
- Duration of therapy (d)	5.4 [±12.1] (0) [0–5]	2.7 [±5.2] (0) [0–3.3]	6.5 [±8.6] (3) [0–10.5]	0.209
CPAP	15 (60)	18 (56)	22 (71)	0.007
- Duration of therapy (d)	7.1 [±13] (1) [0–5]	9.7 [±18.2] (1) [0–5.6]	16.5 [±20] (8) [0–25.5]	0.011
O_2_max under respiratory support (FiO_2_)	0.69 [±0.35] (1) [0.33–1]	0.64 [±0.34] (0.65) [0.21–1]	0.79 [±0.29] (1) [0.63–1]	0.066
**Neonatal complications**				
ROP total	9 (36)	7 (22)	17 (55)	<0.001
- ROP I	1 (4)	5 (16)	1 (3)	0.099
- ROP II	6 (24)	1 (3)	11 (35)	<0.001
- ROP III	2 (8)	1 (3)	5 (16)	0.059
IVH total	1 (4)	0	9 (29)	<0.001
- IVH I	1 (4)	0	3 (10)	0.074
- IVH II	0	0	4 (13)	0.004
- IVH III	0	0	2 (6)	0.063

Table 2 shows descriptive neonatal outcome for the different groups of cerclages. Hospitalization, respiratory outcome and neonatal complications are shown for all preterm neonates admitted to neonatal intensive care unit (NICU). Data are shown as “n (%)”or “mean [±SD] (median) [IQR]

^a^ One neonate died after admission

^b^ Respiratory support includes continuous positive airway pressure (CPAP, n,%) and mechanical ventilation

E-/V-/LBW = extremely/ very/ low birth weight infant, ROP = retinopathy of prematurity, IVH = intraventricular hemorrhage, w = weeks, d = days.

### Descriptive maternal characteristics and pregnancy outcome (Table 1)

**Fig 1:**
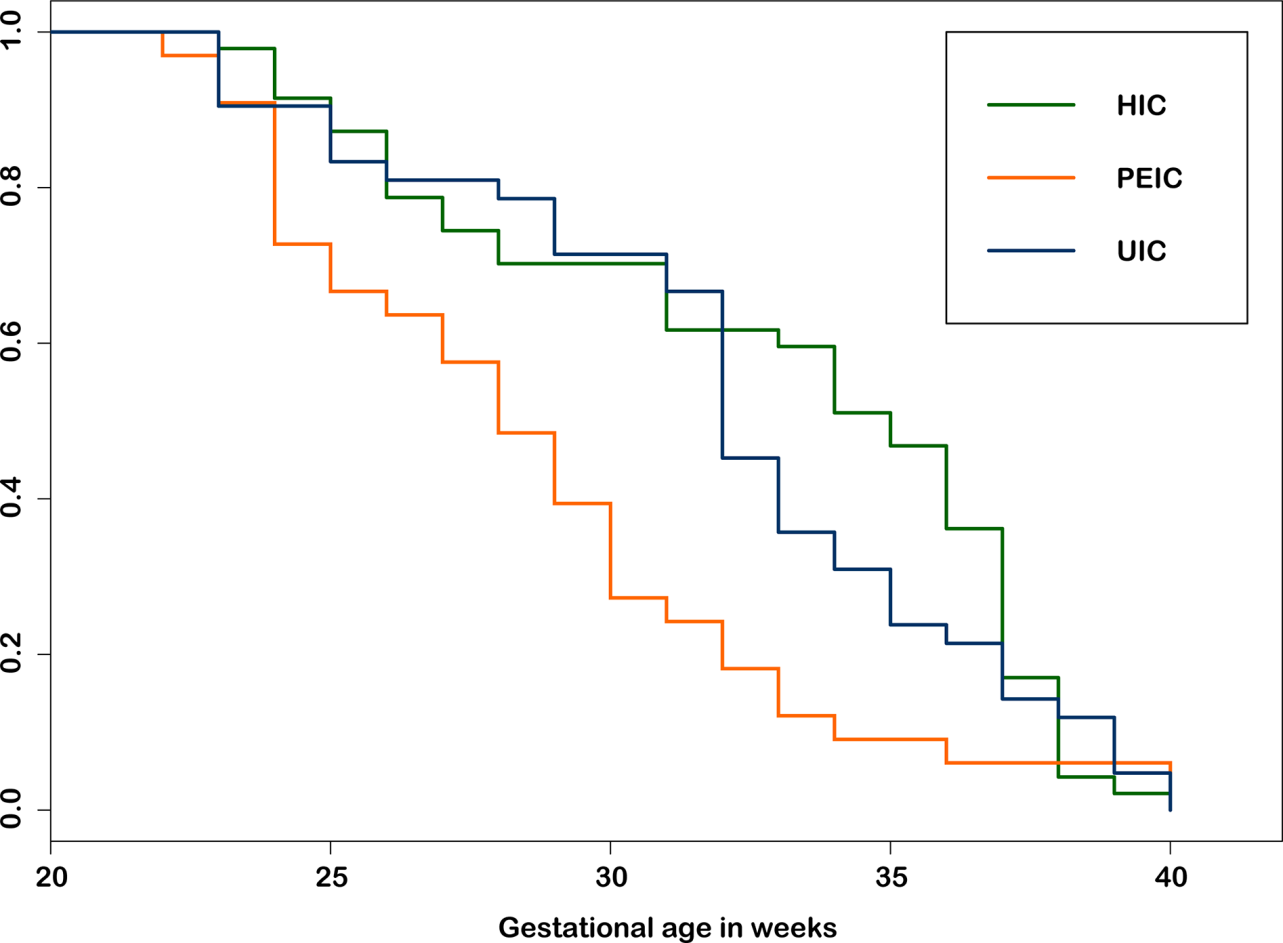
Proportion undelivered in different types of cerclage. Fig 1 displays the gestational age at delivery for the different groups of cerclage. There is significant difference of the Kaplan-Meier estimators of the different types of cerclage (Log-rank test, p = 0.003). HIC and UIC differ significantly from PEIC, however there is no significant difference between UIC and HIC (HIC vs. PEIC: p = 0.00132; UIC vs. PEIC: p = 0.00864; HIC vs. UIC: p = 0.509). To control the family-wise error rates for comparing the different distributions, p-values were adjusted for multiple comparisons by Bonferroni correction.

Overall the study population showed a heterogenic risk profile. Consistent with the different indications, the median GA at placement of cerclage varied significantly (p = 0.002). PEIC were delivered significantly earlier than UIC and HIC, whereas there was no significant difference for the time of delivery between UIC and HIC (see Fig 1). In seven cases labor started before 23+0 weeks (before fetal viability) and infants were not admitted to neonatal intensive care unit (NICU). These cases were excluded from the statistical analysis.

Maternal morbidity and mortality was overall low. Cervical laceration was observed in five cases due to preterm uterine contractions. No case of preeclampsia or severe maternal complications (e.g. thrombosis, pulmonary embolism) were observed during antenatal, delivery or postpartum period.

### Descriptive neonatal outcome (Table 2)

Neonatal outcome was assessed for all preterm neonates (GA < 37+0 weeks, n = 94, of which n = 30 of the HIC-group (n = 26 admitted to NICU, n = 1 died during stay), n = 33 of the UIC-group, n = 31 of the PEIC-group). Neonates after PEIC showed impaired outcome with a significant difference for duration of mechanical ventilation, ROP, IVH, duration of stay at NICU and live birth rate. This difference is in line with our expectations as emergency cerclages target a different risk group than HIC or UIC.

### Statistical analysis

#### a) Neonatal outcome <1500 g (Fig 2)

To compare neonatal outcome and to screen for potential causative perinatal risk factors we performed frequency matching on birth weight categories in the subgroup of neonates <1500 g. Statistical analysis confirmed no significant difference in birth weight (BW) between all four groups (mean [±SD] in grams: BW_HIC_ = 880 [±204], BW_UIC_ = 904 [±290], BW_PEIC_ = 849 [±281], BW_control_ = 870 [±167]; ANOVA, p = 0.72). There was a significant overall difference for GA at delivery (mean [±SD] in weeks: GA_HIC_ = 26 [±2], GA_UIC_ = 26 [±3], GA_PEIC_ = 26 [±3], GA_control_ = 26 [±2]; ANOVA, p = 0.038) with a significant difference between controls and UIC only (p = 0.042). The three groups of cerclage differed neither for GA nor for BW (p = 0.351; p = 0.251 respectively, see Table 3).

**Fig 2:**
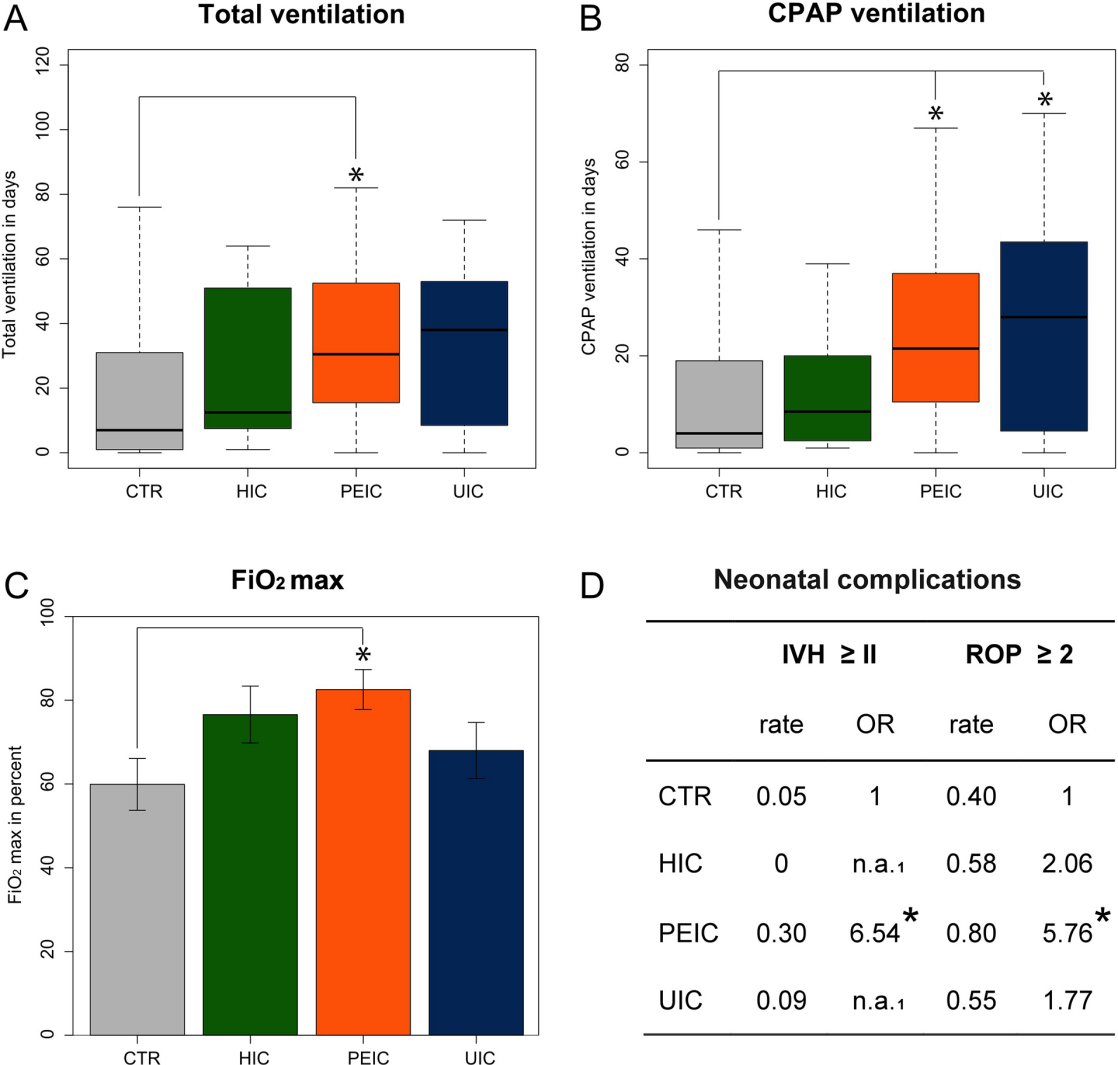
Statistical analysis of neonatal outcome <1500 g. Fig 2 shows outcome of neonates <1500 g for different types of cerclage as well as for birth weight matched controls (n_control_ = 373; n_PEIC_ = 20; n_UIC_ = 10, n_HIC_ = 12). **A-C** Neonates after PEIC show significantly impaired respiratory outcome when compared to corresponding controls (Total ventilation, p = 0.01; CPAP ventilation (continuous positive airway pressure), PEIC: p = 0.01; UIC: p = 0.04; Maximal fraction of inspired oxygen (FiO_2_ max), p = 0.01 bars represent means). **D** Neonates after PEIC are also at higher risk for severe neonatal complications (intraventricular hemorrhage, IVH ≥ II, OR 6.54, 95% CI: 2.11–18.23, p = 0.0007; retinopathy of prematurity, ROP ≥ 2, OR 5.76, 95% CI: 2.04–20.98, p = 0.0028). ^**1**^ due to low case numbers, odds ratios are not applicable; * indicates significant values, p<0.05.

**Table 3:** Perinatal parameters for neonatal subgroup <1500 g.

Parameter	VLBW cerclage groups				
Maternal parameters	HIC (n = 12)	UIC (n = 10)	PEIC (n = 20)	*P*	
PPROM^a^	6 (50)	4 (40)	9 (45)	0.985	
Reason for preterm delivery:^b^					
- contractions	7 (58)	8 (80)	11 (55)	0.395	
- bleeding	0	2 (20)	3 (15)	0.297	
- pathological CTG	3 (25)	0	6 (30)	0.158	
- Clinical signs of chorioamnionitis^c^	4 (33)	3 (30)	9 (45)	0.671	
Delivery mode: C-Section	12 (100)	10 (100)	16 (80)	0.088	
**Infection-associated parameters**					
- CRP levels at delivery (mg/l)^d^	39.4 [±22.8] (37.8) [36.1–50.8]	7.6 [±9.0] (4.2) [2.0–11.2]	44.8 [±36.6] (33.8) [12.0–68.0]	0.015	
- WBC at delivery (x10^9^/L)^d^	12.9 [±4.3] (15.4) [9.7–16.0]	12.5 [±3.7] (11.8) [9.0–16.1]	15.0 [±6.0] (13.0) [11.6–20.0]	0.376	
- Placental pathologic evaluation performed	10 (83)	10 (100)	19 (95)	0.280	
- Placental pathologic evaluation: Histologic chorioamnionitis	3 (30)	3 (30)	13 (68)	0.048	
**Prenatal treatment**					
- Antibiotic treatment	12 (100)	11 (100)	19 (95)	0.569	
- RDS prophylaxis (corticosteroids)	12 (100)	10 (100)	20 (100)	1	
- Tocolysis (fenoterol, i.v.)	7 (58)	6 (60)	17 (85)	0.177	
**Neonatal parameters**					
Birth weight (g)	880 [±204] (860) [795–1008]	904 [±290] (715) [640–1148]	849 [±281] (735) [670–1079]	0.251	
Gestational age (w)	26 [±2] (26) [25–27]	26 [±3] (25) [23–27]	26 [±3] (25) [24–28]	0.351	
1 min APGAR	5.5 [±1.6] (6) [4.8–6.3]	6.4 [±1.6] (6.5) [6–7.3]	4.7 [±1.8] (4) [3–6]	0.059	
5 min APGAR	7.3 [±0.8] (7) [7–7.3]	7.5 [±1.2] (7.5) [6.8–8.3]	6.9 [±1.3] (7) [6–7.5]	0.428	
10 min APGAR	8.2 [±0.8] (8) [8–8.3]	8.1 [±1.1] (8.5) [7.8–9]	7.6 [±1.20] (8) [7–8]	0.231	
PHA	7.30 [±0.05] (7.32) [7.29–7.34]	7.32 [±0.02] (7.33) [7.31–7.33]	7.31 [±0.08] (7.33) [7.26–7.36]	0.841	
PHV	7.32 [±0.06] (7.35) [7.31–7.35]	7.36 [±0.03] (7.36) [7.34–7.38]	7.35 [±0.08] (7.36) [7.30–7.41]	0.862	
BE	-4.3 [±2.9] (-5.0) [-6.1- -3.7]	-0.4 [±1.4] (-0.5) [-1.2- -0.6]	-3.7 [±3.6] (-2.9) [-5.5- -1.5]	0.035	
CRIB-Score	7.3 [±4.9] (8.5) [2.8–11.5]	5.4 [±5.7] (3) [1.8–8.3]	9.3 [±4.4] (10) [7–12]	0.122	

Table 3 shows perinatal parameters for the three groups of cerclage in neonates <1500 g.

^a^ PPROM: Preterm premature rupture of membranes not associated with surgical procedure of cerclage

^b^ Multiple reasons for preterm delivery can apply per case

^c^ Clinical signs of chorioamnionitis is defined as: antenatal maternal fever (Temp ≥100.4°F) plus either leukocytosis (white blood cell count >15x10^9^/L), fetal tachycardia (baseline fetal heart rate >160bpm), fundal or uterine tenderness to palpation, foul-smelling amniotic fluid

^d^ CRP (C-reactive protein) levels and WBC (white blood cell) counts were measured within 12 hours before delivery; CRIB-Score = clinical risk index for babies, PHA/ PHV = umbilical cord arterial/ venous blood pH, BE = base excess, w = weeks, g = grams. Data are shown as “n (%)”or “mean [±SD] (median) [IQR].

**Respiratory outcome:**The overall respiratory outcome of neonates after PEIC was greatly impaired as compared to the group of birth weight matched control neonates with same GA. Infants after PEIC required significantly longer respiratory support with a mean additional 16.6 d (mean [±SD]; PEIC: 35.2 [±24.8]; Ctr: 18.6 [±24.0]; ANOVA, p = 0.0051; Tukey’s test, p = 0.015). The mean respiratory support via CPAP was significantly longer for neonates after PEIC (+12.4 d) (mean [±SD]; PEIC: 25.1 [±20.3]; Ctr: 12.7 [±16.9]; ANOVA, p = 0.0013; Tukey’s test, p = 0.011) and also 13.9 d greater for UICs (mean [±SD]; 26.6 [±23.3], Tukey’s test, p = 0.044). In line with our findings for respiratory support, our data showed a significantly greater maximal fraction of inspired oxygen (FiO_2_ max) under respiratory support for infants after PEIC as compared to controls (mean [±SD]; PEIC: 0.83 [±0.24]; Ctr: 0.60 [±0.32]; ANOVA, p = 0.0051; Tukey’s test, p = 0.010), further stressing the overall compromised respiratory outcome after PEIC.

**Neonatal complications:**Deducible from our findings for respiratory outcome, neonates after PEIC also showed a significantly higher rate of ROP (Kruskal-Wallis test, p = 0.0024). As ROP 1 is not associated with visual impairment we looked at the rate of ROP ≥ 2. Neonates after PEIC showed a higher rate of ROP ≥ 2 of 80% as compared to 40% for controls without cerclage and an odds ratio of 5.76 (Chi-squared test: p = 0.0028; OR 5.76 with [95% confidence interval [CI]: 2.04–20.98]). Our data does not support an overall significant difference for IVH (Kruskal-Wallis test: p = 0.093), however high significance for clinically relevant IVH ≥ II (Chi-squared test: p = 0.0007) with a 30% rate for neonates after PEIC as compared to 5% for controls (OR 6.45 with [95% CI: 2.11–18.23]).

#### b) Perinatal parameters for neonatal subgroup <1500 g (Table 3)

Infection or chronic inflammation play a major role in the pathogenesis of preterm birth and are associated with impaired neonatal outcome. We thus screened for infection-associated perinatal differences and circumstances around delivery in our study population.

Clinical signs of CAM were causative for preterm delivery in 45% of cases in the PEIC subgroup as compared to UIC (30%) and HIC (33%). Almost all patients received prenatal antibiotic treatment. CRP levels differed significantly (high in PEIC and HIC, p = 0.015). White blood cell counts within 12 hours before delivery were not significantly higher in PEIC pregnancies (p = 0.376).

Placental pathological evaluation was performed for almost all cases. The rate of histological CAM was significantly higher in PEIC placentas (68%) as compared to UIC and HIC (both 30%) (p = 0.048).

Short-term postpartal neonatal parameters showed a trend to lower 1’, 5’ and 10’ minute APGAR scores in PEIC neonates. Direct respiratory indicators for neonatal hypoxia such as cord pH did not differ between the groups of cerclage, which might be explained by the overall high rate of cesarean section. The CRIB score (Clinical Risk Index for Babies) as a tool for assessing direct postnatal adaptation with a high positive predictive value for neonatal morbidity and mortality is also higher in PEIC neonates, however not significantly (p = 0.122).

## Discussion

Preterm birth is the single most important cause of neonatal morbidity and mortality. Therefore potential treatment options are urgently needed [11]. However, potential adverse effects of obstetrical interventions need to be considered carefully especially with respect to intermediate and long-term neonatal outcome. In this study we evaluated neonatal outcome after different types of cervical cerclage (HIC, UIC, PEIC) and performed statistical analysis for a subgroup of neonates <1500 g. The scope of our study is not the indication and effectiveness of cerclage as such, but to identify potential harmful effects on the neonate.

HIC and UIC: When considering placement of cerclage in women with previous preterm birth or late miscarriage, the potential management is either to perform HIC or to observe these women closely with serial ultrasound scans and to perform an UIC in case the cervix shortens. Berghella et al. showed no difference for the two strategies and therefore concluded that serial ultrasound scans might be sufficient to monitor these pregnancies safely and additionally avoid unnecessary treatment [12]. The groups of UIC and HIC thus greatly overlap and expectedly outcome is similar in our study as well (see Figs 1 and 2, Table 3).

PEIC: There is limited data on neonatal outcome after emergency cerclage. Althuisius et al. compared emergency cerclage (PEIC) with bed rest versus bed rest alone and found better outcome in cases of cerclage [10]. In our study the cerclage to delivery interval was 43 days with a mean GA at delivery of 28 weeks compared to 54 days and 30 weeks. However, Althusius et al. described outcome for an administration of cerclage at 22.2 weeks (mean), whereas in our trial the surgery was done one week earlier.

Non-cerclage control group: Stoll et al. published detailed data for outcome of VLBW neonates in a large retrospective cohort analysis [9]. Our control of VLBW neonates showed similar outcome parameters as the data of 2000 neonates collected by the NICHD [9] (duration of hospitalization: median = 68 d vs. 63 d, Stoll et al., proportion requiring mechanical ventilation: 44% vs. 40%, Stoll et al.). Differences in clinical outcome despite similar birth weight or hospitalization might be due to different clinical management or national standards (e.g. rate of IVH ≥ II: 5% vs. 11%, Stoll et al., ROP ≥ 3: 9% vs. 3% Stoll et al.). The rate of multiples does not differ in between our study population and data from the NICHD [9].

In a Cochrane review Alfirevic et al. showed a significant reduction of preterm birth before 37, 34 and 28 weeks in women treated with cerclage at risk of recurrent preterm birth [4]. Interestingly, the prolongation of pregnancy was not associated with a significant reduction of neonatal morbidity and mortality [4]. Thus it is crucial to highlight potential adverse effects of cerclage.

Our study reveals impaired neonatal outcome for PEIC in neonates <1500 g. In particular respiratory outcome (longer total ventilation, CPAP ventilation, higher FiO_2_max) and rate of severe neonatal complications (rate of IVH ≥ II, ROP ≥ 2) differ greatly from birth weight matched neonates without maternal cerclage and same GA (see Fig 2). We propose infection and/or inflammation to play a potential role in the pathogenesis (e.g. significantly higher rate of CAM after PEIC as compared to UIC and HIC and significantly higher CRP-levels, see Table 3). Whereas the predictive value of CRP-levels in diagnosing CAM or an intrauterine inflammation is low [5,6], histologically diagnosed CAM gives a valid idea of the prenatal intrauterine inflammatory milieu [5,13]

Preterm cervical changes resemble an inflammatory-like reaction [14]. Placing a cerclage in such an environment might trap the inflammatory environment, which threatens fetal development. The presence of a cerclage in patients with premature rupture of membranes (PROM) further increases the risk of intra-uterine infection and affects neonatal outcome [15,16]. In our study group the rate of preterm PROM (not associated with the surgical procedure) was high in all groups of cerclage (45% PEIC, 40% UIC, 50% HIC, see Table 3). Patients with preterm PROM were admitted and treated with antibiotics according to the hospital standards. As the rate of PROM was high in all groups of cerclage this only insufficiently explains the adverse outcome of neonates after PEIC. It should also be noted that PROM may not be the cause but the consequence of CAM [17].

Histologically diagnosed CAM exceeds the prevalence of clinical CAM [18], which can also be shown in our analysis of PEIC pregnancies (clinical signs of CAM: 45%, histologic CAM: 68%; see Table 3). Subclinical CAM and inflammation not associated to infection may account for this difference [13]. Such an intrauterine inflammation, which can also occur without rupture of membranes and without the presence of microbes in the amniotic fluid, is associated with fetal inflammatory response syndrome (FIRS) resulting in severe neonatal complications [5,7,8]. In particular, IVH and ROP are closely linked to CAM [18], which coincides with our data (see Fig 2).

However, our study harbors many potential confounding variables, which have to be taken into account.

We included an equal fraction of about 10% twin pregnancies in all three subgroups and the control group. The recent Cochrane analysis of cerclage in twin pregnancies shows potentially impaired outcome after UIC in twins, however there is insufficient data to conclude about the outcome of PEIC twin pregnancies [19]. Many other studies include twins as well as singletons, as case numbers are extremely limited [10,20,21]. Berghella correctly demands RCTs in twin pregnancies to clarify the matter [22].

As stated in the results, follow-up data of 6 cases of cerclage placement (4 HIC, 2 UIC) was lost (patients delivered in a different clinic) and cases were excluded from the analysis. In small study populations the exclusion due to insufficient data might represent a selection bias.

Some women in the PEIC group had previous preterm births and second trimester losses and did not receive HIC (see Table 1), as they presented first in our clinic with already dilated cervix and/ or bulging membranes. This history indicated risk might be a potential confounder with a selection bias towards cases with higher risk for adverse outcome in the PEIC group.

Further, the standard prenatal treatment in between the different groups of cerclage did not differ (see Table 3), besides a trend towards a higher rate of tocolysis (fenoterol i.v.) treatment in the PEIC group. A current Cochrane analysis states that betamimetics delay delivery from 48 hours to up to 7 days without reducing the incidence of respiratory distress syndrome and other adverse outcome parameters [23]. During the study period, the use of progestogens was not established in our clinic.

The delivery mode has been described as an independent risk factor for IVH or periventricular leukomalacia (PVL) in neonates [24,25], other studies fail to show this correlation [26]. Especially in VLBW infants data from large trials did not confirm the link between vaginal delivery and IVH [27]. However, the controversy remains and the lower rate of CS in PEICs (80%), as compared to UIC and HIC (100%) might be a confounding variable when looking at IVH as a read-out parameter for severe neonatal complications. On the contrary, CS is associated with impaired respiratory outcome as compared to vaginal delivery [28], further highlighting the impaired respiratory outcome of our PEIC neonates.

Due to the retrospective study design and the small sample size we did not perform adjustments for body-mass-index, calendar year or other possible variables that might affect outcome parameters. This further stresses the need for large multicenter studies in order to be able to control for the numerous confounders [22].

In conclusion, our study indicates that neonates <1500 g after PEIC show impaired outcome. Intrauterine inflammation is an established risk factor for neonatal morbidity and mortality and may play a causal role in our study population. The diagnosis of subclinical intrauterine infection or inflammation is however difficult as valid clinical or biochemical indicators are missing [5]. This study adds controversy to the question whether to prolong or to deliver, however larger case-numbers or RCTs would be needed to give final answers.
